# Mutations in RZF1, a zinc-finger protein, reduce magnesium uptake in roots and translocation to shoots in rice

**DOI:** 10.1093/plphys/kiad051

**Published:** 2023-02-22

**Authors:** Natsuko I Kobayashi, Hiroki Takagi, Xiaoyu Yang, Ayako Nishizawa-Yokoi, Tenta Segawa, Tatsuaki Hoshina, Takayuki Oonishi, Hisashi Suzuki, Ren Iwata, Seiichi Toki, Tomoko M Nakanishi, Keitaro Tanoi

**Affiliations:** Graduate School of Life Sciences, The University of Tokyo, 1-1-1 Yayoi, Bunkyo-ku, Tokyo 113-8657, Japan; Faculty of Bioresources and Environmental Sciences, Ishikawa Prefectural University, 1-308 Suematsu, Nonoichi, Ishikawa 921-8836, Japan; Graduate School of Life Sciences, The University of Tokyo, 1-1-1 Yayoi, Bunkyo-ku, Tokyo 113-8657, Japan; Institute of Agrobiological Sciences, National Agriculture and Food Research Organization (NARO), 3-1-3 Kannondai, Tsukuba 305-8604, Japan; Faculty of Bioresources and Environmental Sciences, Ishikawa Prefectural University, 1-308 Suematsu, Nonoichi, Ishikawa 921-8836, Japan; Graduate School of Life Sciences, The University of Tokyo, 1-1-1 Yayoi, Bunkyo-ku, Tokyo 113-8657, Japan; Center for Education and Research of Community Collaboration, Utsunomiya University, Utsunomiya 321-8505, Japan; National Institutes for Quantum and Radiological Science and Technology, 4-9-1 Anagawa, Inage-ku, Chiba 263-8555, Japan; Cyclotron and Radioisotope Center (CYRIC), Tohoku University, 6-3 Aramaki Aza-Aoba, Aoba-ku, Sendai 980-8572, Japan; Institute of Agrobiological Sciences, National Agriculture and Food Research Organization (NARO), 3-1-3 Kannondai, Tsukuba 305-8604, Japan; Faculty of Agriculture, Ryukoku University, 1-5 Yokotani, Seta Oe-cho, Otsu, Shiga 520-2194, Japan; Graduate School of Nanobioscience, Yokohama City University, 22-2 Seto, Yokohama, Kanagawa 236-0027, Japan; Graduate School of Life Sciences, The University of Tokyo, 1-1-1 Yayoi, Bunkyo-ku, Tokyo 113-8657, Japan; Hoshi University, 2-4-41 Ebara, Shinagawa-Ku, Tokyo 142-8501, Japan; Graduate School of Life Sciences, The University of Tokyo, 1-1-1 Yayoi, Bunkyo-ku, Tokyo 113-8657, Japan

## Abstract

Magnesium (Mg) homeostasis is critical for maintaining many biological processes, but little information is available to comprehend the molecular mechanisms regulating Mg concentration in rice (*Oryza sativa*). To make up for the lack of information, we aimed to identify mutants defective in Mg homeostasis through a forward genetic approach. As a result of the screening of 2,825 M2 seedlings mutated by ion-beam irradiation, we found a rice mutant that showed reduced Mg content in leaves and slightly increased Mg content in roots. Radiotracer ^28^Mg experiments showed that this mutant, named low-magnesium content 1 (LMGC1), has decreased Mg^2+^ influx in the root and Mg^2+^ translocation from root to shoot. Consequently, LMGC1 is sensitive to the low Mg condition and prone to develop chlorosis in the young mature leaf. The MutMap method identified a 7.4-kbp deletion in the LMGC1 genome leading to a loss of two genes. Genome editing using CRISPR-Cas9 further revealed that one of the two lost genes, a gene belonging to the RanBP2-type zinc-finger family that we named *RanBP2-TYPE ZINC FINGER1* (*OsRZF1*), was the causal gene of the low Mg phenotype. OsRZF1 is a nuclear protein and may have a fundamental role in maintaining Mg homeostasis in rice plants.

## Teaser

The zinc-finger protein RZF1 is required for magnesium uptake in roots and transport to shoots according to a rice mutant screening study.

## Introduction

Magnesium (Mg) is the second most abundant intracellular cation in plant cells. It exists predominantly as a complex form with cellular components with a unique binding-affinity for each (Cowan 2002), and the concentration of free Mg^2+^ in the cytosol is about 0.3 mM (Maguire and Cowan 2002; Gout et al. 2014). In the cellular components, Mg is efficiently incorporated with various metabolic cycles by serving a catalytic role and allosteric effects (Pierce and Reddy 1986).

In response to limited Mg availability in the environment, a decrease in the phloem loading of sucrose is one sign of Mg deficiency. The rationale of this phenomena is that H^+^-ATPases, which consume ATP energy, are sensitive to Mg deficiency, and the proton-motive force created by H ^+^ -ATPases drives sucrose transporters that mediate phloem sucrose loading (Geiger 2011). After that, the overaccumulation of photoassimilates, and finally the onset of leaf interveinal chlorosis, have been observed in this order in bean (*Phaseolus vulgaris*) (Cakmak et al. 1994), sugar beet (*Beta vulgaris*) (Hermans et al. 2005), and Arabidopsis (*Arabidopsis thaliana*) (Hermans and Verbruggen 2005; Ogura et al. 2020). Characteristics of Mg deficiency compared to other mineral deficiencies include a high susceptibility of young mature leaves. Whole plant iodine staining revealed that starch accumulates preferentially in the most recently expanded young leaves (Hermans et al. 2004; Hermans and Verbruggen 2005; Kobayashi et al. 2013; Ogura et al. 2020). These leaves can already function as an energy source with increasing photosynthetic activity while the Mg concentration drastically decreases during leaf expansion. An Mg concentration below 1–2 mg g^−1^ is a benchmark for the onset of chlorosis in plants (Hermans et al. 2013). At about the same time as or after chlorosis appears, leaf growth rates start to be reduced (Hermans and Verbruggen 2005) most probably due to reduced carbon partitioning and photosynthesis. The degree of the growth retardation and the time of its appearance will vary depending on the age of the plant materials used in the experiment (Hermans et al. 2013), as they can relate to Mg storage amounts and Mg retranslocation capability in plant tissues.

Magnesium is known to be mobile in many plant species (Hocking 1994; Maillard et al. 2015). In rice (*Oryza sativa*) plants, the concentration of Mg^2+^ in phloem sap was reported to be 12 mM (Fukumorita et al. 1983), which is apparently higher than the Mg^2+^ concentration in the cytosol. Active Mg retranslocation includes the removal of Mg from chlorophyll structures by the Mg dechelatase *STAY-GREEN* SGR (Peng et al. 2019), but other details of the retranslocation process including phloem Mg^2+^ loading are not yet known. Magnesium uptake and xylem transport is also properly controlled. The concentration of Mg^2+^ in rice xylem sap is concentrated compared to the external solution when the Mg^2+^ concentration of the external solution is less than 3 mM (Tanoi et al. 2011). In *Arabidopsis*, MAGNESIUM RELEASE4 (MGR4) and MGR6, are responsible in this mechanism as xylem loaders in the root stele (Tang et al. 2022a, 2022b; Meng et al. 2022), and METAL TOLERANCE PROTEIN10 (MTP10) acts as a xylem unloader in the shoot parenchyma cells in vascular bundles (Ge et al. 2022). Mutations in these transporters cause modification in Mg content in the whole plant. Mmembers of CorA superfamily also function in Mg^2+^ dynamics (Schock et al. 2000; Li et al. 2001; Gebert et al. 2009). For the Mg^2+^ uptake system, Arabidopsis MITOCHONDRIAL RNA SPLICING 2/MAGNESIUM TRANSPORTER 6 (AtMRS2–4/MGT6) (Mao et al. 2014), OsMGT1/OsMRS2-2 (Chen et al. 2017), and CorA-LIKE ZntB MAGNESIUM TRANSPORTER 1 (OsCZMT1) (Lim et al. 2022) are suggested to be involved, although the mechanistic basis for their function has not fully been elucidated. Keeping magnesium stored in the vacuole is another important strategy for surviving environmental fluctuations. AtMRS2-1/MGT2 (Conn et al. 2011; Tang et al. 2022a, 2022b), AtMRS2-5/MGT3 (Conn et al. 2011), AtMRS2-10/MGT1 (Tang et al. 2022a, 2022b), and MGR1 (Tang et al. 2022a, 2022b) are the tonoplast localized transporters participating in this process. Loss of these molecules results in unbalanced Mg distribution among *Arabidopsis* organs. Additional Mg^2+^ might be stored in the endoplasmic reticulum (ER) (Oda et al. 2016), where AtMRS2-7/MGT7 (Gebert et al. 2009) and possibly AtMRS2-4/MGT6 (Oda et al. 2016) also seem to be involved in Mg^2+^ transport. Apart from the ion transporters, the root endodermal barrier contributes to Mg homeostasis in leaves, given that a mutation in SHENGEN3 leads to Mg overaccumulation in *Arabidopsis* (Pfister et al. 2014) and *Brassica rapa* (Alcock et al. 2021).

These previous works are gradually revealing the molecules that act as valves in the Mg ion distribution circuit. Now, it is necessary to identify more components involved in the Mg homeostasis to understand the whole picture. In this study, we physiologically and genetically characterized a rice mutant with low Mg content in leaves, which we isolated from a screening of an ion-beam mutagenized M2 population. The mutant, named low-magnesium content 1 (LMGC1), showed reduced Mg^2+^ influx and impaired root-to-shoot Mg transport from the early stages of growth. The causative mutation of the low Mg phenotype of LMGC1 was found through MutMap + analysis, leading to the identification of the causal gene encoding RanBP2-type zinc-finger protein.

## Results

### Disturbance in Mg distribution in LMGC1

**Figure 1:**
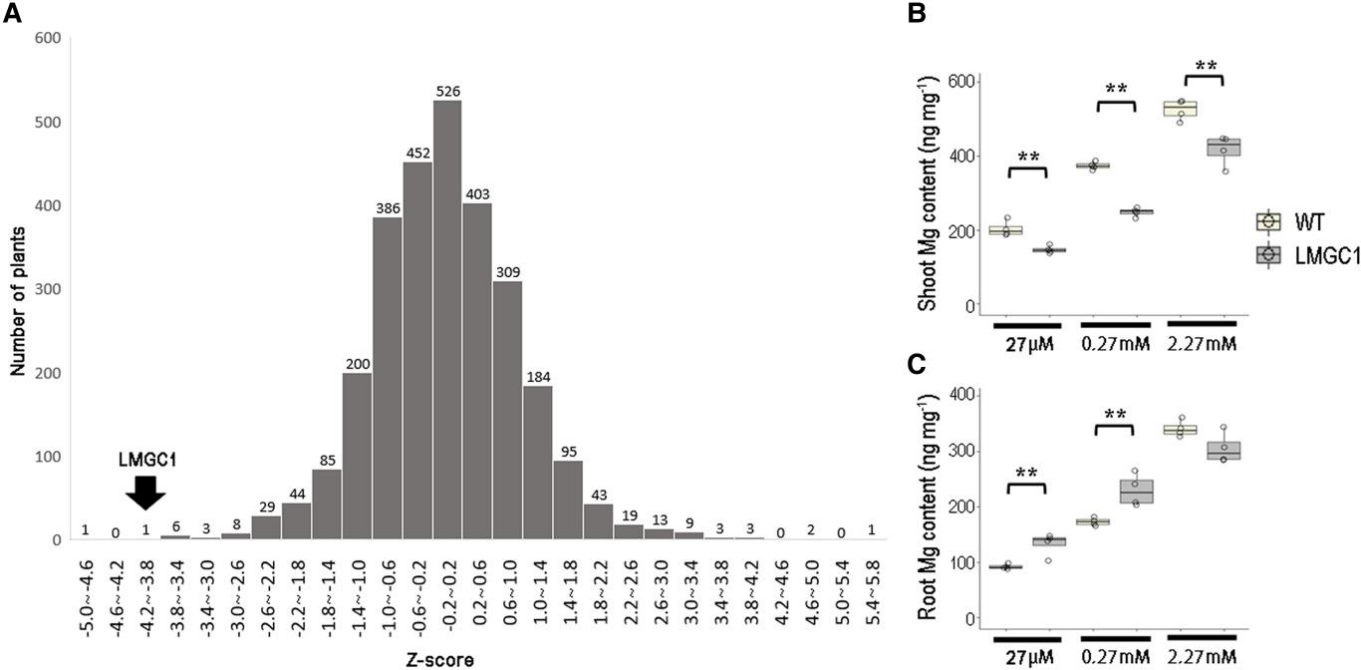
Identification of the low-magnesium rice mutant LMGC1. A) Histogram showing the distribution of the shoot Mg/K content ratio of the ion-beam irradiated M2 population in the first screen, where plants were grown with 54 μM Mg. The Z-score of LMGC1 was −4.10. Magnesium content in shoots B) and roots C) of wild-type and LMGC1 mutant plants (*n* = 4). Following cultivation in 0.5 mM CaCl_2_ solution, rice plants were grown for 1 wk in nutrient solution containing either 27 μM (low Mg), 0.27 mM (control), or 2.27 mM (high Mg) of Mg^2+^. Asterisks indicate significant differences between the wild-type (WT) and LMGC1 mutant (**P < 0.01, Student's *t*-test). Center line, median; box limits, upper and lower quartiles; whiskers, 1.5 × interquartile range; points, each sample data.

A total of 2,825 M2 plants were screened according to Mg/K content ratio in the shoot. Due to space limitations in the plant growth chamber, approximately 50 plantlets were screened at a time. The z-score of each plant in each group was calculated, and then a histogram of the z-score of the whole population was produced (Fig. 1A). Then, the plants with a Mg/Ca ratio similar to their Mg/K ratio were preferentially selected, and those showing abnormal growth were excluded. Consequently, one mutant with the most distinctly decreased Mg content in shoots under low Mg condition was identified and named LMGC1. The initial Z-score of LMGC1 was −4.10 (Fig. 1A). Consistent with what we had observed during the screening process, the shoot Mg content of LMGC1 plants grown with a nutrient solution for one week was decreased compared to that of the wild-type Koshihikari regardless of the Mg^2+^ concentration in the nutrient solution (Fig. 1B). The largest decrease was 35%, which was observed under the control condition (Fig. 1B). In the roots, however, LMGC1 was found to accumulate more Mg than the wild-type under control and low Mg conditions (Fig. 1C). As a result, the distribution of Mg in the shoot and the root was greatly altered in LMGC1.

### Growth retardation observed under low Mg condition

**Figure 2:**
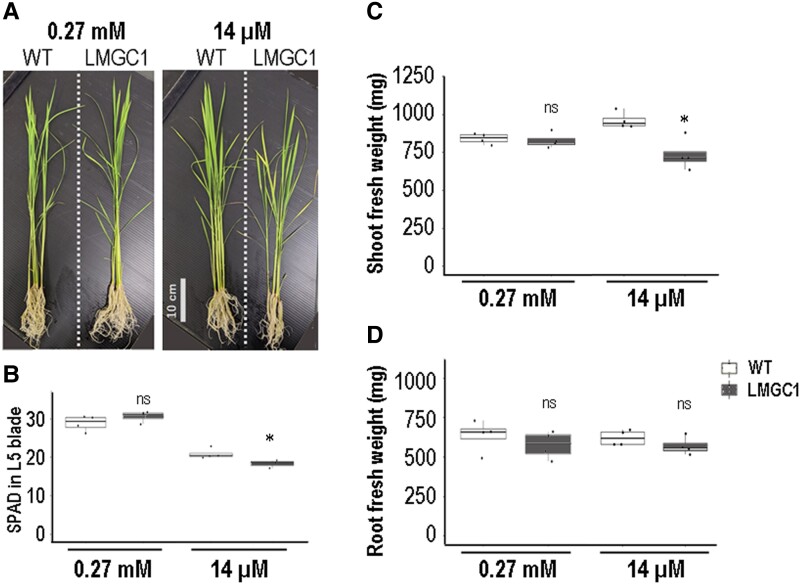
Phenotypes of LMGC1 after cultivation in nutrient solution containing either 14 μM (low Mg) or 0.27 mM (control) of Mg^2+^ for 22 d. A) Changes in appearance (*n* = 4). B) SPAD value at the leaf blade of the 5th leaf (*n* = 3–4, 3 technical replicates each). C) The fresh weight of the shoot (*n* = 4). D) the fresh weight of the root (*n* = 4). In B), (C), and D) “ns” indicates not significant (*P* > 0.05), whereas the asterisk indicates significant difference (*P* < 0.05) between the wild-type and LMGC1 mutant (Student's *t*-test). Center line, median; box limits, upper and lower quartiles; whiskers, 1.5 × interquartile range; points, each sample data.

The effect of the low Mg content in shoots on the plant growth was examined. After 22 d, LMGC1 grew similarly to the wild-type Koshihikari unless enough Mg was supplied (Fig. 2A). Under a low Mg condition, the color of young mature leaf tips were lighter for LMGC1 compared with the wild-type, and the leaves of LMGC1 were beginning to wither (Fig. 2A). Accordingly, SPAD value in the 5th leaf, which was one of the mature leaves, was lowered (Fig. 2B), and the shoot biomass was decreased (Fig. 2C) in LMGC1 compared with in the wild-type under the low Mg condition. In contrast, root growth was not reduced significantly in LMGC1 (Fig. 2D).

### Magnesium uptake kinetics and translocation from roots to shoots

**Figure 3:**
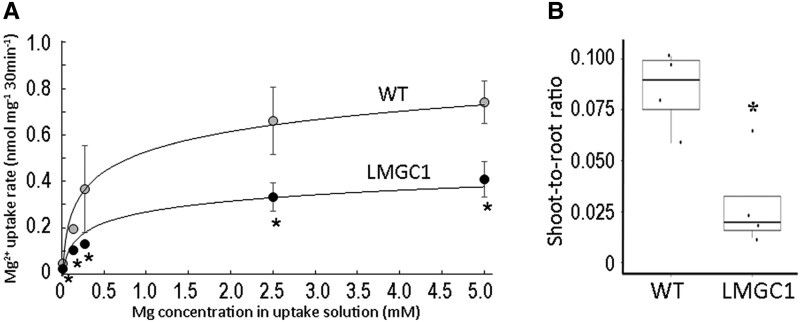
Kinetic analysis of Mg^2+^ uptake and transport determined by a ^28^Mg tracer experiment using wild-type (WT) and LMGC1 plants grown in nutrient solution (0.27 mM Mg^2+^) for 1 wk following cultivation in CaCl_2_ solution. A) Mg^2+^ uptake rate in nutrient solution with various Mg^2+^ concentrations (15–5000 μM) (*n* = 4) (*P < 0.05, Student's *t*-test). Error bars indicate SD. B) ^28^Mg shoot-to-root ratio in the uptake experiment using 0.25 mM Mg^2+^ solution (*n* = 4) (*P < 0.05, Student's *t*-test). Center line, median; box limits, upper and lower quartiles; whiskers, 1.5 × interquartile range; points, each sample data.

To understand how Mg distribution in LMGC1 is altered, the Mg^2+^ dynamics in the seedlings grown under control condition for 1 wk were analyzed using the radiotracer ^28^Mg. The Mg^2+^ uptake rate in LMGC1 was found to be reduced by half in any Mg^2+^ concentration in the uptake solution (Fig. 3A). Focusing on the experiment with 0.25 mM Mg^2+^ concentration in the uptake solution, the shoot-to-root ratio of ^28^Mg was less than one-third in LMGC1 compared to that in the wild-type (Fig. 3B).

### Modified Mg^2^+ dynamics in LMGC1 found the day after transplanting to the nutrient solution

**Figure 4:**
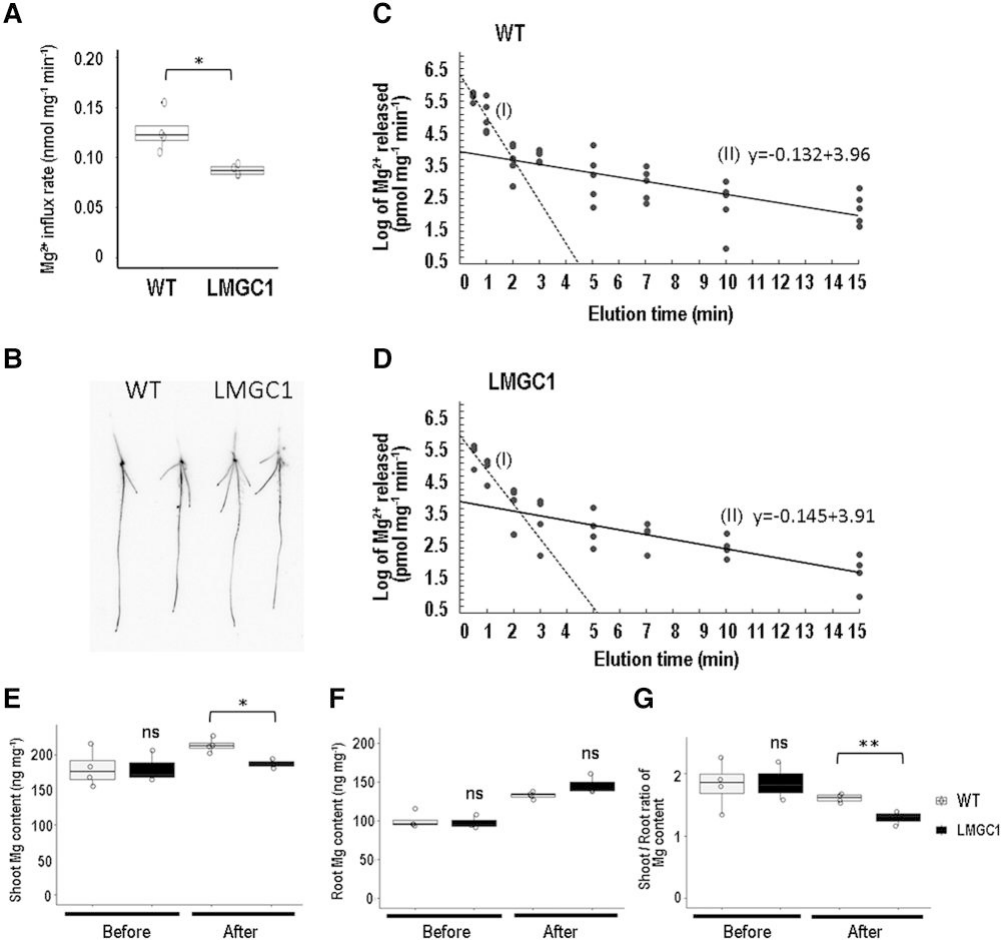
Magnesium ion flux and distribution in the wild-type (WT) and LMGC1 mutant cultivated in nutrient solution (0.27 mM Mg^2+^) for 1 d following cultivation in CaCl_2_ solution. A) Magnesium influx determined based on the ^28^Mg radioactivity after 20 s absorption (*n* = 4). B) ^28^Mg radioactivity images of the seedlings after 20 s absorption (*n* = 2). C-D) Time-course of Mg^2+^ elution in the wild-type C) and LMCG1 mutant D). Semi-logarithmic plots were made using 4–5 seedlings to construct the efflux curves. The efflux rate (nmol mg^−1^ min^−1^) was determined based on the y-intercept of the second linear regression line (II) with a more gradual slope. The *R^2^* values between individual values in each measurement were 0.56–0.94 for WT and 0.77–0.98 for LMGC1. The first steep line I) represents the release of Mg^2+^ from the root surface and intercellular apoplastic spaces. The experiments were conducted twice, and the results from the first experiment are presented. E, F) Magnesium content in the shoots E) and the roots F) of the seedlings before transplantation to the nutrient solution (0.27 mM Mg^2+^) and 24 h after transplantation (*n* = 4). G) The Mg shoot-to-root ratio. Asterisks indicate significant differences between the wild-type and LMGC1 mutant (**P < 0.01, *P < 0.05), whereas “ns” indicates there is no significant difference (*P* > 0.05) (Student's *t*-test). For A), E), F), and G), Center line, median; box limits, upper and lower quartiles; whiskers, 1.5 × interquartile range; points, each sample data.

With the result that the amount of Mg^2+^ uptake in 30 min was reduced in LMGC1, we tried to distinguish whether the influx had decreased, or the efflux had increased. Based on the ^28^Mg signal intensity after 20 seconds' absorption, the Mg^2+^ influx rate (nmol mg^−1^ min^−1^) was calculated to be 0.126 for the wild-type and 0.088 for LMGC1 (Fig. 4A). The distribution image of ^28^Mg in the seedlings showed that the signal intensity in LMGC1 was reduced throughout the root, but the signal distribution was not different from that of the wild-type (Fig. 4B). The efflux rate (nmol mg^−1^ min^−1^) was calculated to be 0.052 for the wild-type (*R^2^* = 0.56–0.94), and 0.050 for LMGC1 (*R^2^* = 0.77–0.98) based on the y-intercept of the linear regression (Fig. 4, C and D). The experiment was repeated twice, and the results of the second experiment were 0.051 and 0.060 for the wild-type and LMGC1, respectively. The difference in efflux rate between the wild-type and LMGC1, if any, was found to be obviously smaller than the difference in the influx rate.

Given that the decrease in the influx rate in LMGC1 was detected as early as the day after transplanting into the nutrient solution, we proceeded to test the possibility that the decrease in influx rate preceded the disturbance in Mg distribution. The Mg content in the shoots of LMGC1 was comparable with that of the wild-type before transplanting to the nutrient solution, but it was lower than that of the wild-type the day after transplanting into the nutrient solution (Fig. 4E). In the roots, although there was no statistically significant difference, Mg content of LMGC1 tended to increase compared with that in the wild-type after transplanting to the nutrient solution (Fig. 4F). Consequently, the shoot/root ratio of Mg content was significantly lower in LMGC1 than in the wild-type already the day after transplanting to the nutrient solution (Fig. 4G).

### Large deletion in chromosome 1

**Figure 5:**
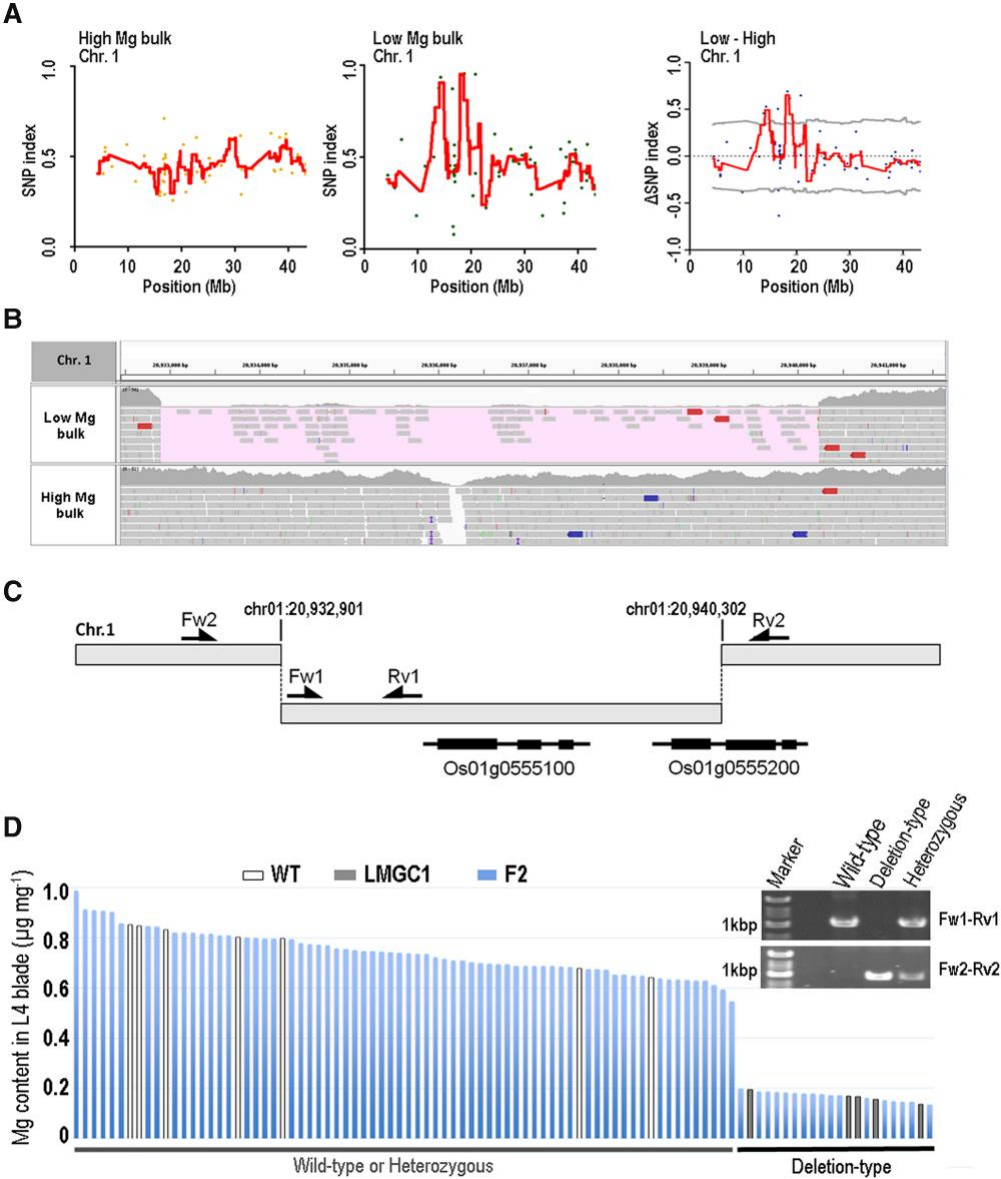
Identifying the causal mutation for the low Mg content of LMGC1. A) The identification of a candidate genomic region on chromosome 1 based on MutMap + analysis. The dots represent a SNP-index value at a given SNP position. The regression lines represent the sliding window average SNP-index values with a 2 Mb interval and 50 kb increment. The two horizontal lines in the delta (SNP-index) graph are the 95% confidence interval of SNP-index value under the null hypothesis of a randomly bulked DNA sample. B) Visualization of the aligned sequence reads around 20 Mb on chromosome 1 with Integrative Genomics Viewer. The region covering few sequence reads in only low bulk sample is highlighted. C) Schematic illustration of the physical relationship between the large deletion and the localizing tow genes, Os01g0555100 and Os01g0555200. The primers used for detecting the genotype in the large deletion are indicated with arrows (Fw1-Rv1 and Fw2-Rv2). D) The relationship between the leaf Mg content and genotype in the F2 progenies. The primer pairs, Fw1-Rv1 and Fw2-Rv2, amplify a 1-kb PCR product in the wild-type allele and a 0.9-kb mutation allele from the large deletion, respectively. Individuals showing PCR products from both primer pairs have a heterozygous genotype in this locus.

F2 progenies were developed by crossing LMGC1 with the wild-type Koshihikari. Among 90 individuals of the F2 plants, 10 individuals containing the highest and lowest concentrations of Mg were used in the MutMap + approach as high and low bulk samples, respectively (Abe et al. 2012; Fekih et al. 2013). First, the “Koshihikari-reference” was developed by replacing nucleotides of the publicly available reference genome for rice “IRGSP-1.0” (https://rapdb.dna.affrc.go.jp/download/irgsp1.html) with those of Koshihikari nucleotides at all homozygous SNP positions. Next, NGS short reads obtained from high and low bulk samples were aligned to the “Koshihikari-reference”. The SNP-index values representing the frequency of mutant alleles in the bulk sample were calculated genome-wide. Finally, a sliding window analysis (SWA) for calculating average SNP-index values, with a 2-Mb window and 50-kb step sizes, was applied (Supplemental Fig. S1). In this analysis, statistically significant peaks were detected as candidate genomic regions within chromosome (Chr)1 (13,100,000–15,900,000 and 16,950,000–20,300,000) (Fig. 5A). Although two SNP positions, 18,733,068 and 20,776,574, showing high SNP-index values (>0.95) specifically in low bulk samples were identified, both SNPs caused no amino acid substitutions. Therefore, to identify large structural variation, we compared the sequence depth between bulk samples within the above candidate regions on Chr1. As a result, 7.4 kb at 20,932,901–20,940,302 on Chr1 had few short reads in specifically low bulk samples (Fig. 5B), making this 7.4 kb a candidate deletion for the low Mg concentration in LMGC1. By this 7.4 kb deletion, Os01g0555100 and Os01g0555200 are completely and partially excluded from the genome (Fig. 5C). To verify that the 7.4 kb deletion is associated with the low Mg phenotype of LMGC1, we confirmed the association between leaf Mg content and genotype in 80 individuals of F2 progeny derived from a different F1 individual from the one used for MutMap + analysis. All 17 individuals with a homozygous 7.4 kb deletion confirmed by PCR had lower Mg contents compared with homozygous and heterozygous WT individuals, indicating that this deletion is a strong candidate for the causal mutation for the low Mg phenotype of LMGC1 (Fig. 5D).

### Identification of the causal gene for LMGC1

**Figure 6:**
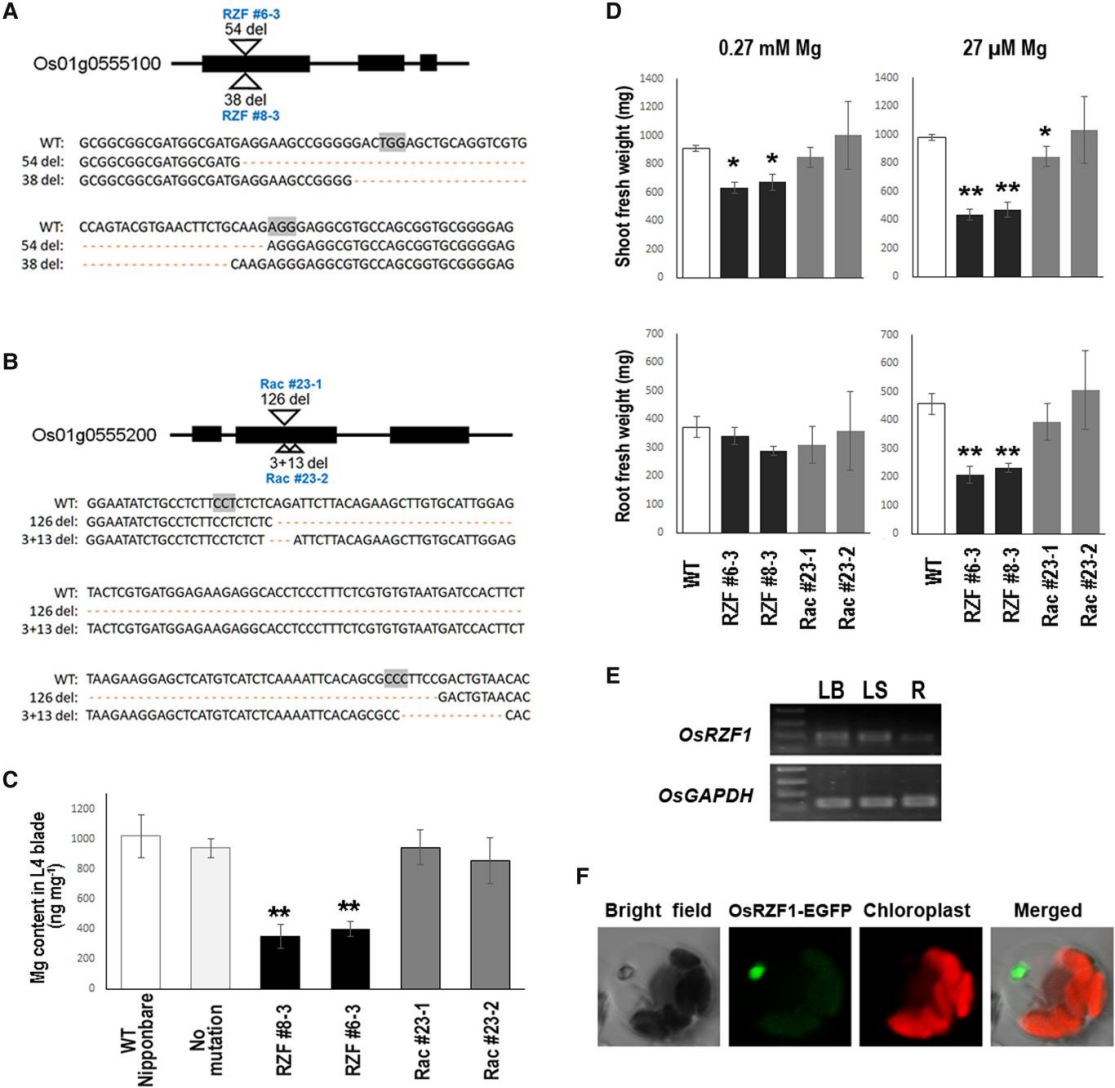
Identification of the *OsRZF1* gene through analysis of CRISPR/Cas9-mediated mutant lines, and determination of *OsRZF1* expression and localization. A) RZF #6-3 and RZF #8-3 have deletion mutations in the first exon of the Os01g0555100 gene. A part of the coding sequence (13–111 bp in the WT) is presented. B) Rac #23-1 and Rac #23-2 have deletion mutations in the second exon of the Os01g0555200 gene. A part of the coding sequence (277–437 bp in the WT) is presented. The PAM sequences used for the genome editing are highlighted. C) Magnesium content in the blade of the 4th leaf of the mutant lines grown under control condition. Wild-type Nipponbare plants and agrobacterium-infected plants that gained antibiotic resistance but failed to cause any mutation in the targeted gene (no mutation) were used as the reference (*n* = 4–7). Error bars indicate SD. D) Shoot and root biomass of plants grown under 27 μM (low Mg) or 0.27 mM (control) of Mg^2+^ for 22 d (*n* = 3). Error bars indicate SD. In C) and D), the mutants with asterisks are significantly different from the wild-type Nipponbare (*P < 0.05, **P < 0.01, Student's *t*-test). E) Expression of *OsRZF1* gene analyzed by RT-PCR. *OsGAPDH* was used as a reference. LB; leaf blade, LS; leaf sheath, R; root. F) Transient expression assay of OsRZF1-EGFP fusion protein in rice protoplast.

The first candidate, Os01g0555100, was previously identified as one of the stress repressive zinc-finger proteins (Huang et al. 2008) belonging to the RanBP2-type zinc-finger (RZF) protein family. The second candidate, Os01g0555200, was predicted to be a Asp/Glu racemase (Rac) family protein based on its amino acid sequence. To validate the causal gene for LMGC1, we generated mutants using CRISPR/Cas9-targeted mutagenesis in the Nipponbare rice cultivar, and investigated the Mg content in the leaf. The mutants RZF #6–3 and RZF #8–3 had deletions of 54 and 38 bp in Os01g0555100, respectively (Fig. 6A). Both deletion mutations resulted in the loss of one of the three zinc-finger domains, and the 38 bp deletion further caused a frameshift. Rac #23-1 was a mutant with a 126-bp deletion, and Rac #23-2 had a 3-bp and a 13-bp deletion (Fig. 6B). As a result of the 126-bp deletion, 42 residues out of a total of 329 residues in the Rac protein were lost. The mutation identified in Rac #23-2 led to a frameshift causing an alteration in the amino acid sequence including a loss of the asparagine and threonine residues functioning as a binding site for the substrate (Ahn et al. 2015). Measurement of Mg content in the leaves of these mutant lines revealed that mutations in the *RZF* gene, but not the *Rac* gene, can reproduce the low Mg phenotype of LMGC1 (Fig. 6C).

The biomass of the mutants RZF #6-3 and RZF #8-3 was less than half that of WT Nipponbare under the low Mg condition (Fig. 6D, Supplemental Fig. S2), which further supported the idea that the *RZF* gene is the causal gene for LMGC1. The slightly lighter shoot weight of the RZF mutants compared to that of the WT Nipponbare even under the control condition and the significant reduction in root weight under the low Mg condition (Fig. 6D), which were not observed in LMGC1 (Fig. 2D), may imply that there are some cultivar differences in the importance of this gene. We named this gene *OsRZF1* and performed expression analysis to explore its potential role. The gene was expressed in both leaves and roots, with a higher expression level in the leaves (Fig. 6E). Transient expression assay using rice leaf protoplasts showed EGFP fluorescence localized to the nucleus (Fig. 6F), indicating that OsRZF1 is a nuclear protein.

## Discussion

Previously, ion-beam mutagenesis in rice had achieved identification of *Oryza sativa NATURAL RESISTANCE-ASSOCIATED MACROPHAGE PROTEIN 5* (*OsNRAMP5*) as the gene responsible for grain Cd content after screening of 2,592 M2 plants (Ishikawa et al. 2012), *Oryza sativa SALT OVERLY SENSITIVE 2* (*OsSOS2*) for low Cs rice after screening of 2,710 M2 plants (Ishikawa et al. 2017), and here we successfully isolated a low Mg rice mutant. It is of interest that the causative gene for LMGC1, which had the most distinctly reduced Mg concentration among 2,825 M2 plants screened, was not a Mg transporter gene. Based on the ^28^Mg radiotracer experiment, it was found that a reduced influx (Fig. 4A) and unchanged efflux (Fig. 4, C and D) resulted in the lowered Mg uptake in LMGC1 (Fig. 3A). We also found a quantitative relationship between the influx and efflux of Mg^2+^ in the rice root. The calculated efflux rate in the wild-type was 0.052 nmol mg^−1^ min^−1^ (Fig. 4C), and the difference between this and the influx rate, 0.126 nmol mg^−1^ min^−1^, corresponded to the uptake rate, namely, 0.074 nmol mg^−1^ min^−1^. It is suggested that Mg homeostasis in rice roots is maintained by a futile cycling system in which nearly half of the Mg^2+^ entering the root cells is discharged and only the other half is acquired. This idea is comparable with the very close values of Mg^2+^ efflux and influx in excised onion (*Allium cepa*) roots (Macklon and Sim 1976).

In ion uptake and translocation, the main molecules may be distinct, or there may be some molecules involved in both. In the former case, the ion uptake from the rhizosphere into the root outer cells can be mediated by influx transporters such as the potassium transporter HIGH-AFFINITY K^+^ 5 (HAK5) (Rubio et al. 2000; Qi et al. 2008) and manganese transporter OsNRNAMP5(Sasaki et al. 2012), whereas xylem loading is implemented by the efflux transporters functioning in ion exclusion from cells in the neighborhood of the xylem such as the potassium STELAR K^+^ OUTWARD RECTIFIER (SKOR) channel (Gaymard et al. 1998), H^+^/K^+^ antiporter NITRATE TRANSPORTER 1; 5 (NRT1; 5) (Li et al. 2017), and manganese transporter OsMTP9 (Ueno et al. 2015). The lack of any one of these transporters substantially reduces the above-ground content of the ions that the transporter was carrying (Gaymard et al. 1998; Nieves-Cordones et al. 2010; Sasaki et al. 2012; Ueno et al. 2015). Thus, we assumed that the decreased shoot Mg content in LMGC1 would be associated with some molecules functioning in primary Mg^2+^ transport. However, xylem Mg^2+^ loading is mediated by efflux-type transporters in *Arabidopsis* (Meng et al. 2022), suggesting that the systems for uptake and translocation are distinct. We thought that if it was the Mg^2+^ influx function that was directly impaired in LMGC1, it can be reasonable to assume that the reduction in influx would have occurred prior to the reduction in Mg^2+^ translocation. However, in the present experiment, both phenotypes were observed just the day after transplanting into the nutrient hydroponic solution (Fig. 4, A and G). Therefore, it is impossible to determine at present whether one of the two phenotypes, Mg^2+^ influx and translocation, is the primary cause or whether both occur independently downstream of OsRZF1.

Investigations on CRISPR/Cas9-mediated mutants demonstrated that the Mg homeostasis in rice requires the *OsRZF1* gene, expressed in both leaves and roots. The protein encoded by *OsRZF1* contains three RanBP2-type zinc-finger domains. The consensus sequence of this domain is W-X-C-X_2–4_-C-X_3_-N-X_6_-C-X_2_-C (C4-type), which generally recognizes ssRNA (Loughlin et al. 2009; Nguyen et al. 2011). In the plant RZF phylogenetic tree, *OsRZF1* is in the same clade as rice *STRESS REPRESSIVE ZINC FINGER 1* (*SRZ1*) (Huang et al. 2008) and Arabidopsis *STRESS ASSOCIATED RNA-BINDING PROTEIN 1* (*SRP1*) (Xu et al. 2017). Both *SRZ1* and *SRP1* expressions can be responsive to ABA, salt, and cold treatments (Huang et al. 2008; Xu et al. 2017). In ABA signaling, the ABA-PYL-PP2C-SnRK2s pathway plays the central role to control transcription factors and ion channels downstream of ABA signaling pathway (Ng et al. 2014). During osmotic stress, several potassium transporters and anion channels have been identified as the ion channels controlled by SnRK2 proteins to facilitate stomatal responses (Osakabe et al. 2013). SRP1 has been suggested to be involved in this ABA pathway through modulating ABSCISIC ACID-INSENSITIVE 2 (ABI2), one of the PP2C proteins of negative regulators, by directly binding to the 3′UTR of *ABI2* mRNA to reduce its stability (Xu et al. 2017). It should be also noted that ABI2 can interact with and activate SOS2, which is a member of SnRK3 protein family required for the Na^+^ efflux activity of SOS1 (Ohta et al. 2013). In addition, there is another RZF protein participating in ABA signaling pathway. The protein is SUPPRESSOR OF ABI3-5 (SUA), which harbors one zinc-finger domain and is required for the proper splicing of *ABI3* gene (Sugliani et al. 2010). Although SUA belongs to another clade than the clade to which SRP1 belongs (Gipson et al. 2020), it is noteworthy that the main components of ABA signaling use multiple RZF proteins as modulators.

The functions of other C4-type RZF proteins, including rice SRZ1, at the molecular level are currently unknown. However, functions for regulating gene turnover and/or gene stability can be assumed for other proteins belonging to this clade (Gipson et al. 2020). For OsRZF1, transcriptomic data in the public database RiceXPro indicates that expression of *OsRZF1* is also ABA sensitive, letting us derive the hypothesis that the ABA signal pathway controls some Mg^2+^ channels downstream and OsRZF1 modulates them by interacting with some components of ABA signaling, just like how SRP1 works. Another possibility is that OsRZF1 is a splicing-related protein required for the maturation of some molecules participating in Mg^2+^ transport, apart from ABA signaling. The zinc-finger domain of SUA protein is shown to have binding-affinity to the nucleotide sequence GGA (De Franco et al. 2019), which is found in several hexamer motifs of potential exonic splicing enhancer in *Arabidopsis* (Pertea et al. 2007). To understand the mode of action of OsRZF1 further, we need to identify the genes to which OsRZF1 binds.

In this study, we identified OsRZF1 as a factor highly influential for the Mg^2+^ transport system. In particular, OsRZF1 is required for growth under low Mg conditions. We suggest that a C4-type RZF protein plays an important role in mineral homeostasis in rice.

## Materials and methods

### Plant growth conditions

Rice (*Oryza sativa*. L.) “Koshihikari” plants were germinated in pure water and grown further on plastic mesh floating in 0.5 mM CaCl_2_ solution (Ca solution) for 2–3 d, then transferred to half strength Kimura B nutrient solution (Kobayashi et al. 2018). Magnesium concentration in the nutrient solution was modified by adding MgSO_4_ or substituting MgSO_4_ for Na_2_SO_4_ according to the experimental subjects. The nutrient solutions were refreshed every 2–3 d. The plant incubator was set to 28°C with a 10-h/14-h light/dark photoperiod.

### Elemental analysis

The shoots of ion-beam irradiated rice Koshihikari M2 plants (Ishikawa et al. 2012) grown hydroponically with 54 μM Mg for 2 wk were harvested for screening. The remaining roots and the basal part of shoots, including nodes and unelongated internodes, were cultivated to grow new shoots and further harvest M3 seeds. After the identification of the low Mg mutant LMCG1, the Koshihikari wild-type and LMGC1 mutant (M4 plants) were grown for 1 wk in the nutrient solution containing either 27 μM (low Mg), 0.27 mM (control), or 2.27 mM (high Mg) of Mg^2+^ and harvested. Rice seedlings were also sampled just before transplanting them into the nutrient solution and one day after transplanting. The “Nipponbare” cultivar wild-type and genome-edited mutant lines generated by the method shown in the later section were also used for elemental analysis. They were grown in the control nutrient solution for 2 wk and the blade of the 4th leaf was sampled. Shoots, roots, or leaf blades were separately weighed and digested completely in 69% (v/v) nitric acid at 90°C using a closed vessel digestion procedure. Sample solutions were analyzed with Inductively Coupled Plasma-Mass Spectrometry (NexION, PerkinElmer) after being diluted appropriately with ultrapure water. The quantification of the method was verified using the certificated reference material (NCS DC73349, China National Analysis Center).

### Growth assay

Rice plants were grown under either 14 or 27 μM Mg (low Mg), or 0.27 mM Mg (control) conditions for 22 d until the 7th leaf was matured. The SPAD value of the 5th leaf at 5 cm from the tip of the leaf blade was determined as the average of 3 measurements using a chlorophyll meter (SPAD-502 plus, Konica Minolta), and the fresh weight of shoots and roots was measured.

### Magnesium uptake, translocation, and flux analysis using a radiotracer

Carrier-free ^28^Mg with a half-life of 21 h was produced by the ^27^Al(α,3p)^28^Mg reaction in a cyclotron and chemically purified (Tanoi et al. 2013). Wild-type and LMGC1 mutant plants were grown in nutrient solution containing 0.27 mM Mg^2+^ for 1 wk. For the measurement of the Mg uptake rate, the roots of the plants were soaked for 30 min in uptake solution, which was half strength Kimura B nutrient solution with Mg (15-5000 μM) labeled with ^28^Mg. After absorption, the roots were rinsed with ice-cold nutrient solution for 10 min. Then, the remaining solution on the surface was wiped off with a paper towel, and the samples were separated into shoots and roots, weighed, and subjected to gamma-ray counting (Kobayashi et al. 2018). The amount of Mg accumulated in the shoots and the roots during 30 min was added up and divided by the root weight to obtain the Mg uptake rate (nmol mg^−1^ 30min^−1^). Additionally, the translocation of Mg^2+^ from roots to shoots was calculated based on the ^28^Mg concentration ratio of the shoots and the roots.

For the Mg^2+^ flux analysis, Mg^2+^ influx and efflux in the roots were independently measured (Kobayashi et al. 2022). The roots of the plants grown with the nutrient solution with 0.27 mM Mg for 24 h were soaked in the uptake solution containing 0.27 mM Mg labeled with ^28^Mg (4.8 kBq/ml) for 20 s, and then rinsed for 30 s in ice-cold nutrient solution. Weighing and the radioactivity measurement were performed as described above. Based on the Mg amount absorbed in 20 s, the influx rate was determined to be the value converted to the amount absorbed per minute per unit weight of the root (nmol mg^−1^ min^−1^). In addition, the distribution of ^28^Mg in the seedlings after being rinsed was visualized using an imaging plate (BAS IP MS, GE Healthcare) and image reader (FLA-5000, GE Healthcare) (Kobayashi et al. 2013).

To analyze the efflux rate, the seedlings grown in the CaCl_2_ solution were transferred to the same uptake solution that was used in the influx analysis, and left for labeling for 24 h to ensure that all Mg^2+^ taken into the roots from the nutrient solution was uniformly labeled with ^28^Mg. After labeling, the roots of the plants were quickly dipped in the non-labeled nutrient solution to remove the ^28^Mg remaining on the surface, and then soaked in a series of non-labeled nutrient solution for elution of ^28^Mg for 0.5, 0.5, 1, 1, 2, 2, 3, and 5 min. The total duration of elution was 15 min. The amount of Mg^2+^ released from the root per minute per unit weight of the root (pmol mg^−1^ min^−1^) was determined based on the radioactivity in the elution solution and the assumption that the specific radioactivity of Mg^2+^ released from the roots was equal to the uptake solution (Pitman 1971). The basic theory to determine the efflux rate was that of compartmental analysis, established in the 1990s (Lee and Clarkson 1986a, 1986b; Kronzucker et al. 1995). The amount of Mg^2+^ released versus time was plotted in a semi-logarithmic graph, and two linear regressions were fitted to natural logarithms of the rate of Mg^2+^ released. We interpreted that the first steep line represents the release of Mg^2+^ from the root surface and intercellular apoplastic spaces, and that the second line represents the Mg^2+^ release from the cytosol. The y-intercept of the second line represents the rate of Mg^2+^ release from cytosol at time zero, which indicates the efflux rate (pmol mg^−1^ min^−1^).

### Identifying the candidate genomic region of LMGC1

An F2 progeny was developed by a cross between LMGC1 and wild-type Koshihikari. The 90 F2 individuals were grown with the control nutrient solution for 2 wk, and the blade of 4th leaf was used for elemental analysis. Among the F2 plants, DNA from the 10 individuals with the highest and lowest Mg concentrations were pooled to prepare High and Low Mg bulk samples, respectively. The bulked DNA samples were subjected to NGS using a DNBSEQ-G400 instrument (MGI tech) with an insert size of 350 bp, and MutMap + analysis (Fekih et al. 2013) using QTL-seq pipeline version 2 s (Itoh et al. 2019) was carried out. The sequencing dataset was deposited in the DDBJ database (PRJDB14655). The sequence reads were directly applied without any filtering. Alignment was carried out with BWA ver 0.7.17 (Burrows-Wheeler Aligner) software (Li and Durbin 2009) and alignment files were converted to SAM/BAM files using SAMtools ver 1.0 (Li et al. 2009). In this QTL-seq, first, “Koshihikari-reference” was developed by replacing nucleotides of the publicly available reference genome for rice “IRGSP-1.0” (https://rapdb.dna.affrc.go.jp/download/irgsp1.html) with those of Koshihikari nucleotides at all homozygous SNP positions detected by aligning “Koshihikari” sequence reads (DRR237052, DRR237053, DRR237054) to “IRGSP-1.0”. Next, NGS short reads obtained from high and low bulk samples were aligned to “Koshihikari-reference”. In this analysis, spurious SNP positions where wild-type Koshihikari detected SNP in self alignment and both bulk samples detected SNPs showing commonly high and low SNP-index (>0.6 and <0.4) were excluded. After detecting the genomic region harboring contrast SNP-index values between High and Low Mg bulks, we compared the depth of aligned sequence reads between bulk samples. Finally, the genomic regions covering few sequence reads specifically in Low Mg bulk samples were detected as a candidate large deletion for the phenotype of LMGC1. The association between this large deletion and Low Mg content was confirmed by other F2 progeny by PCR with primers designed at flanking regions of the detected deletion (Supplemental Table S1).

### Generation of knock-out mutants

#### Vector construction

The 20-nt annealed oligonucleotide pairs for the target sequences at the 1st exon of RZF (Os01g0555100) or 2nd exon of Rac (Os01g0555200) shown in the Supplemental Table S1 were cloned into the BbsI site of the single guide RNA (sgRNA) expression vector reported previously as pU6gRNA-oligo (Mikami et al. 2015). Two sgRNA expression cassettes targeting RZF (U6::sgRZF-1 and U6::sgRZF-2) or Rac (U6::sgRac-1 and U6::sgRac-2) were amplified by PCR with the primer sets (Supplemental Table S1), tandemly combined and inserted into the AscI site in pZH_OsU6sgRNA _SpCas9 (Endo et al. 2019) by In-Fusion reaction (Takara). CRISPR/Cas9 binary vectors were transformed into *Agrobacterium tumefaciens* EHA105 (Hood et al. 1993) by electroporation (E. coli Pulser, BioRad).

#### Transformation of rice calli

Agrobacterium-mediated transformation was performed as previously described (Toki 1997). Rice “Nipponbare” calli were grown on N6D medium at 33°C for 4 wk and were infected with Agrobacterium harboring the CRISPR/Cas9 binary vector. After 3 d of co-cultivation with Agrobacterium at 23°C on solidified 2N6-AS medium, the calli were washed and transferred to N6D medium containing 25 mg/l meropenem (Fujifilm Wako Pure Chemical Industries) and 50 mg/l Hygromycin B (Fujifilm Wako Pure Chemical Industries) at 33°C for 4 wk. Hygromycin-resistant calli were subjected to HMA assay to identify the callus lines containing mutations at the target site. The callus lines containing mutations at the target site were transferred to a regeneration medium containing 25 mg/l meropenem and cultured at 28°C under a 16 h light:8-h dark condition for 4 wk to obtain the regenerated mutant plants.

#### Heteroduplex mobility assay (HMA)

Genomic DNA was extracted from hygromycin-resistant calli using a one-step DNA extraction protocol (Kasajima et al. 2004). The RZF or Rac loci containing sgRNA target sites were amplified using KOD one PCR master mix (TOYOBO) and the primers listed in the Supplemental Table S1. PCR products were analyzed using MCE-202 MultiNA with a DNA-500 kit (SHIMADZU).

#### Gene expression analysis

Nipponbare plants were grown with control nutrient solution for 1 wk, and the blade and the sheath of the third leaf as well as the root were sampled. RNA was extracted with the use of TRIsure reagent (Bioline), 500 ng of which was used for reverse transcription using SuperScript IV (Invitrogen, USA). The relative amount of accumulation of *OsRZF1* (Os01g0555100) was determined using the *OsGAPDH* gene as a control (Santos et al. 2018) with PCR using KOD one PCR master mix (TOYOBO) (Supplemental Table S1).

#### Protein localization assay

The ORF of the *OsRZF1* gene without the stop codon was amplified (Supplemental Table S1) and cloned to the entry vector with a pENTR™/D-TOPO® Cloning Kit (Invitrogen, USA). Then the 2 × 35 s promoter (Marquès-Bueno et al., 2016) and amplified *OsRZF1* ORF fused with EGFP marker were inserted into the pUC19 plasmid (Takara Bio Inc. Japan) using a Gibson assembly system (New England BioLabs, USA). Rice protoplasts were isolated and transformed according to the method previously reported (Saito et al. 2013). After 18 h, the EGFP fluorescence was detected using Zeiss LSM880 AxioObserver, equipped with an Argon laser as the excitation source, through a 20x/0.8 plan-Apochromat objective lens. EGFP was excited with an Argon laser at 561 nm and an intensity of 0.5% and detected between 597–624 nm with a detector gain value of 900. Images were analyzed and merged by ImageJ (NIH).

#### Statistical analysis

The data of growth parameters, Mg content, and ^28^Mg uptake were analyzed using unpaired Student's *t*-tests to compare the wild-type and the mutants. In the boxplots, the interquartile range with the median are presented. Mean values with standard deviations are presented in the bar charts and the scattergrams.

### Accession numbers

Sequence data from this article can be found in the GenBank/EMBL data libraries under accession numbers AK111255.1; *OsRZF1*, PRJDB14655; genome sequence data used for MutMap + analysis.

## Supplementary Material

kiad051_Supplementary_Data

## Supplemental data

The following materials are available in the online version of this article.

**Supplemental Figure S1**. MutMap + analysis in F2 segregating population derived from a cross between LMGC1 and wild-type Koshihikari.

**Supplemental Figure S2**. Shoot and root dry weights of mutant lines grown under 27 μM (low Mg) or 0.27 mM (control) of Mg^2+^ for 22 d (*n* = 3).

**Supplemental Table S1**. List of primers.
