# Supplementary material for: Mutations in RZF1, a zinc-finger protein, reduce magnesium uptake in roots and translocation to shoots in rice
Source: Plant Physiol. 2023 Feb 22;192(1):342–55. doi: 10.1093/plphys/kiad051 (PMC10152673; doi:10.1093/plphys/kiad051)

Supplemental Table S1.

List of primers.

Primer name	Sequence (5' to 3')	Purpose of use
gRZF-1F	GTTGCGATGAGGAAGCCGGGGGAC	Construction of the sgRNA expression vector targeting RZF
gRZF-1R	AAACGTCCCCCGGCTTCCTCATCG	
gRZF-2F	GTTGAGTACGTGAACTTCTGCAAG	
gRZF-2R	AAACCTTGCAGAAGTTCACGTACT	
gRac-1F	GTTGCTTCTGTAAGAATCTGAGAG	Construction of the sgRNA expression vector targeting Rac
gRac-1R	AAACCTCTCAGATTCTTACAGAAG	
gRac-2F	GTTGGAGCAGTGTTACAGTCGGAA	
gRac-2R	AAACTTCCGACTGTAACACTGCTC	
pZH_AscI OsU6-F	TCTCTAAGCTTGGCGCGCCGGTACCGAGCTCGGATCCAC	Amplification of U6::sgRZF-1 or U6::sgRac-1 to construct CRSIRP/Cas9 vector
U6gRNA-T7seq-R	CTATAGTGAGTCGTATTAAAGCTTATCGATACCGTCGA	
T7seq-U6gRNA-F	ATACGACTCACTATAGGGGGTACCGAGCTCGGATCCAC	Amplification of U6::sgRZF-2 or U6::sgRac-2 to construct CRSIRP/Cas9 vector
pZH_AscI polyT-R	TTACGAATTTCAGGCGCGCCAAGCTTATCGATACC	
RZF HMA-F	AGGTAGATAGAGATGGAGACGAAGG	HMA analysis for the identification of *rzf* mutant
RZF HMA-R	GCGGCTGGCGTAGTTGTT	
Rac HMA-F	TTGGATGCGAGTTCTTCTGC	HMA analysis for the identification of *rac* mutant
Rac HMA-R	GCATGGCAATGCAGCATAC	
Fw1	GAGGAGGTCCATCATTCCAA	Determination of the long deletion in the chromosome 1
Rv1	TGGCCAATACTCATCCCATT	
Fw2	GGAGGCCCCGTTGTTAAATA	
Rv2	CAACAAAAGGAGGAGATCAGA	
RZF-ORF Fw	CACCATGGAGACGAAGGCGGCGGCG	Amplification of the ORF of RZF gene for cloning
RZF-ORF Rv	ATCAACGTAGTATCTGGGGAAATTGC	
RZF RT-PCR Fw	CTTTGCTCGATAACGACCATCAT	Gene expression analysis by RT-PCR
RZF RT-PCR Rv	GATCCAGTCGCCGTTCTTC	
GAPDH RT-PCR Fw	CTGGTATGGCTTTCCGTGTT	
GAPDH RT-PCR Rv	ACCTGTTGTCACCCTGGAAG	

Supplemental Figure S1.

MutMap+ analysis in F2 segregating population derived from a cross between LMGC1 and wild type Koshihikari.
(A) SNP-index plots of low high samples, (B) SNP-index plots of low bulk samples, and (C) ΔSNP-index plots obtained by subtraction of low bulk SNP-index from high bulk SNP-index for the 12 rice chromosomes. Red lines represent the sliding window average of 2Mb interval with 50 kb increment. Gray lines in (C) represent the sliding window average of 2Mb interval with 50 kb increment for the 95% statistical confidence interval under the null hypothesis of randomly bulked DNA sample.

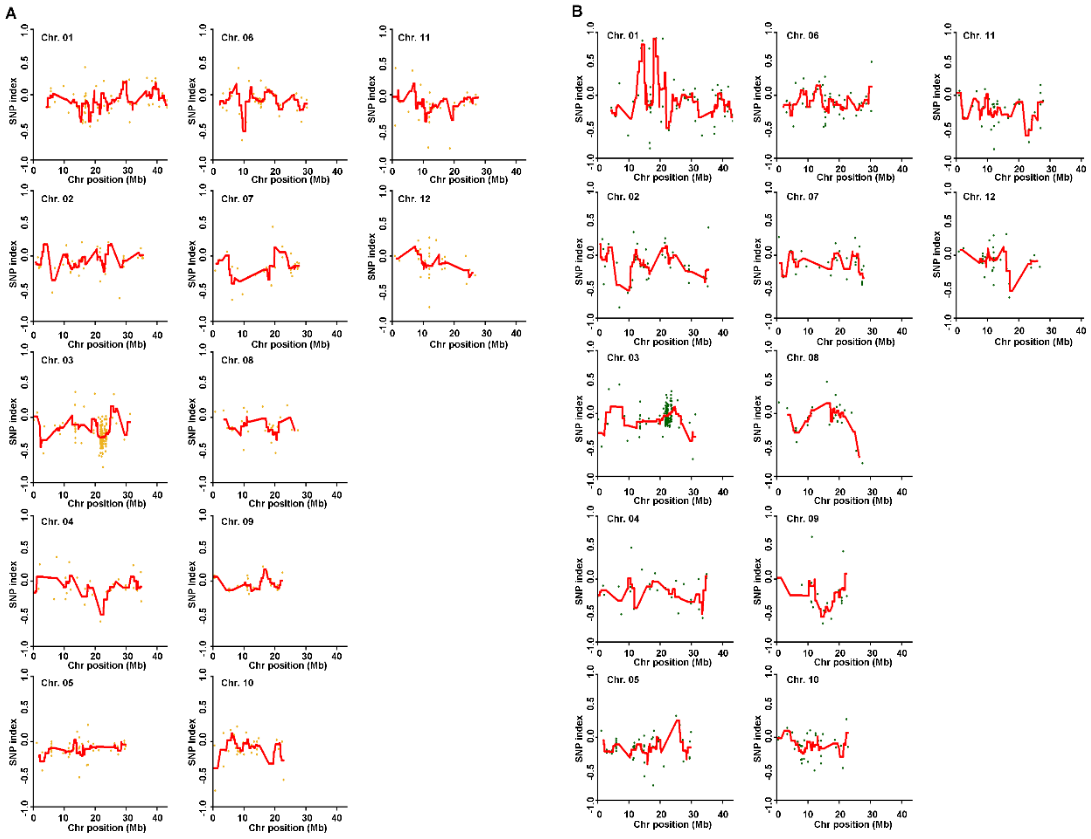

C

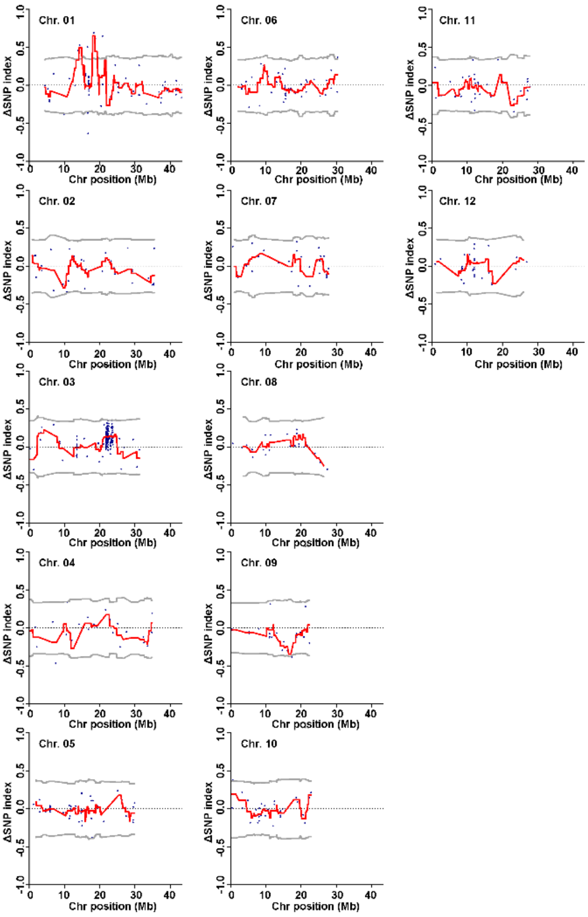
Chr. 01
Chr. 02
Chr. 03
Chr. 04
Chr. 05
Chr. 06
Chr. 07
Chr. 08
Chr. 09
Chr. 10
Chr. 11
Chr. 12
ΔSNP index
Chr position (Mb)

Supplemental Figure S2.

Shoot and root dry weights of mutant lines grown under 27 µM (low Mg) or 0.27 mM (control) of Mg^{2+} for 22 days (n =3). Error bars indicate SD. The mutants with asterisks are significantly different from the wild-type Nipponbare (** $p < 0.01$, Student's t-test). Fresh weight data is presented in Figure 6D.

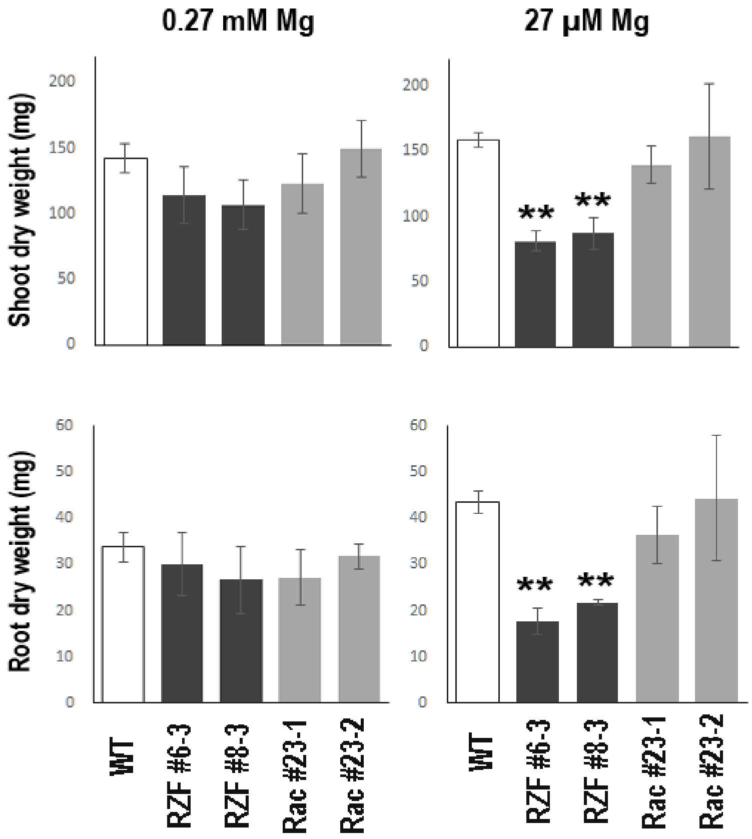

Supplement: kiad051_Supplementary_Data [file kiad051_supplementary_data.pdf]
